# P11 Improving the management of long-term palliative patients on outpatient parenteral antimicrobial therapy (OPAT): a pathway development initiative

**DOI:** 10.1093/jacamr/dlaf239.015

**Published:** 2026-01-05

**Authors:** Shamanth Soghal, Charlene Richards, Katerina Corser, Milind Arolker, James Scriven

**Affiliations:** Department of Geriatric Medicine, University Hospitals Birmingham NHS Foundation Trust, Birmingham, UK; OPAT Service, University Hospitals Birmingham NHS Foundation Trust, Birmingham, UK; OPAT Service, University Hospitals Birmingham NHS Foundation Trust, Birmingham, UK; Supportive and Palliative Care Team, University Hospitals Birmingham NHS Foundation Trust, Birmingham, UK; OPAT Service, University Hospitals Birmingham NHS Foundation Trust, Birmingham, UK; Department of Infection and Tropical Medicine, University Hospitals Birmingham NHS Foundation Trust, Birmingham, UK; School of Infection, Inflammation and Immunology, University of Birmingham, Birmingham, UK

## Abstract

**Background:**

OPAT is evolving to accommodate increasing numbers of patients receiving long-term suppressive treatment. These include individuals with incurable infections or life-limiting conditions where curative therapy is no longer the goal. As OPAT teams increasingly support palliative care scenarios, this shift presents challenges in defining appropriate care goals and has significant implications for resource allocation and service planning.

**Objectives:**

To evaluate current practices in managing patients with life-limiting conditions receiving long-term suppressive antimicrobial therapy via OPAT, and to develop a pathway incorporating palliative care input to support patient-centred decision-making.

**Methods:**

A retrospective review was undertaken of OPAT patients at University Hospitals Birmingham (UHB) who received more than six weeks of antibiotics between January and June 2025. Electronic case notes were reviewed to determine the reason for antibiotic treatment (curative versus suppressive therapy) and whether the patient was identified as having a life-limiting condition, as defined by the Gold Standards Framework (prognosis less than 12 months). Limitations in the data system for identifying palliative patients were also assessed. Stakeholder discussions were conducted with the palliative care team to explore integration into the referral process and further management of patients receiving long-term suppressive therapy.

**Results:**

Between January and June 2025, a total of 306 patients were accepted onto the OPAT service at UHB, of whom 23 received antimicrobial therapy for more than six weeks. Of these 23 patients, seven were classified as receiving suppressive treatment. Within this cohort, treatment goals were often not clearly defined by the referring teams. Patients with complex conditions such as LVAD-related infections (*n*=3), metastatic cancer (*n*=5), and vascular graft infections (*n*=1) were frequently treated for extended periods and experienced multiple readmissions. It was evident that our current data system does not reliably capture patients with life-limiting conditions, making retrospective identification difficult and limiting service evaluation. Discussions with the palliative care team identified opportunities for earlier involvement, particularly at the point of OPAT referral, to clarify treatment intent and align care with patient goals.

**Conclusions:**

Patients with life-limiting conditions receiving long-term suppressive therapy via OPAT represent a unique and growing cohort. Current data systems lack mechanisms to identify these patients early or to support shared decision-making around treatment duration and goals. Early identification and involvement of palliative care can support shared decision-making, clarify treatment goals, and improve patient-centred outcomes.

Proposal for a new pathway: This will include: (i) screening for life-limiting conditions at the point of OPAT referral; (ii) modifying our OPAT database to prospectively clarify treatment intent (curative versus suppressive); (iii) early palliative care referral for patients on suppressive therapy with prognosis less than 12 months; and (iv)improved documentation of patient goals and quality-of-life considerations.

